# Bardoxolone Methyl Prevents Mesenteric Fat Deposition and Inflammation in High-Fat Diet Mice

**DOI:** 10.1155/2015/549352

**Published:** 2015-11-05

**Authors:** Chi H. L. Dinh, Alexander Szabo, Yinghua Yu, Danielle Camer, Hongqin Wang, Xu-Feng Huang

**Affiliations:** ^1^Centre for Translational Neuroscience, School of Medicine, University of Wollongong and Illawarra Health and Medical Research Institute, Wollongong, NSW 2522, Australia; ^2^ANSTO LifeSciences, Australian Nuclear Science and Technology Organisation, Lucas Heights, NSW 2234, Australia

## Abstract

Mesenteric fat belongs to visceral fat. An increased deposition of mesenteric fat contributes to obesity associated complications such as type 2 diabetes and cardiovascular diseases. We have investigated the therapeutic effects of bardoxolone methyl (BARD) on mesenteric adipose tissue of mice fed a high-fat diet (HFD). Male C57BL/6J mice were administered oral BARD during HFD feeding (HFD/BARD), only fed a high-fat diet (HFD), or fed low-fat diet (LFD) for 21 weeks. Histology and immunohistochemistry were used to analyse mesenteric morphology and macrophages, while Western blot was used to assess the expression of inflammatory, oxidative stress, and energy expenditure proteins. Supplementation of drinking water with BARD prevented mesenteric fat deposition, as determined by a reduction in large adipocytes. BARD prevented inflammation as there were fewer inflammatory macrophages and reduced proinflammatory cytokines (interleukin-1 beta and tumour necrosis factor alpha). BARD reduced the activation of extracellular signal-regulated kinase (ERK) and Akt, suggesting an antioxidative stress effect. BARD upregulates energy expenditure proteins, judged by the increased activity of tyrosine hydroxylase (TH) and AMP-activated protein kinase (AMPK) and increased peroxisome proliferator-activated receptor gamma coactivator 1-alpha (PGC-1*α*), and uncoupling protein 2 (UCP2) proteins. Overall, BARD induces preventive effect in HFD mice through regulation of mesenteric adipose tissue.

## 1. Introduction

Obesity is associated with an increased likelihood of chronic diseases such as cardiovascular disease, type 2 diabetes, high blood pressure, and fatty liver disease. It is believed that the mesenteric adipose tissue is important in the pathogenesis of obesity [1–4]. Mesenteric adipose tissue is drained via the portal vein, which delivers fatty acids and proinflammatory cytokines secreted by this tissue to the liver. Studies have shown that this can cause local and systemic inflammation [2] as well as an interruption in insulin action leading to severe insulin resistance in liver [5–7]. Additionally, an increase in mesenteric adipose tissue is linked to inflammation and oxidative damage in vascular tissue and to metabolic syndrome in the gastrointestinal tract and liver [3, 8, 9]. Reports have shown that mesenteric adipose tissue is an independent determinant of metabolic syndromes in humans [10, 11].

Changes in mesenteric fat morphology (adipocyte expansion), an increased level of inflammation and oxidative stress, and reduced energy metabolism are all features of mesenteric fat in obesity. Studies have shown that the mesenteric adipose tissue of high-fat diet (HFD) induced obese mice has an increase in number of large-sized adipocytes [8, 12]. Additionally, obese mice and humans have a greater number of infiltrating macrophages and increased proinflammatory cytokines such as interleukin-1 beta (IL-1*β*) and tumour necrosis factor alpha (TNF-*α*) in mesenteric adipose tissue [8, 13, 14]. The recruitment of anti-inflammatory macrophage phenotype (M2) to inflammatory macrophage phenotype (M1) in mesenteric adipose tissue is linked to colitis, hepatic steatohepatitis, insulin resistance, and development of obesity [15, 16]. Moreover, it has been shown that the increased fat deposition and proinflammatory cytokines in visceral fat depots generate reactive oxidative stress, which in turn escalates inflammation and obesity [17–20].

The positive energy balance that drives obesity is a consequence of impaired energy metabolism. In peripheral tissues, AMP-activated protein kinase (AMPK) is an energy sensor involved in energy metabolism and whole body energy homeostasis via the integration of nutrient, hormone, and stress signals [21–24]. Reduced AMPK activity has been widely observed in obesity* in vivo* and* in vitro* [25, 26]. The reduction of AMPK also reduces energy regulation by peroxisome proliferator-activated receptor gamma coactivator 1-alpha (PGC-1*α*) and uncoupling protein 2 (UCP2) molecules in many peripheral tissues, including mesenteric fat, skeletal muscle, and liver [27–29]. These changes result in increased adiposity, stress, and inflammation [26, 30]. Moreover, the reduction in noradrenergic and adrenergic activity is linked to reduced thermogenesis, increased body weight, and development of obesity [31–33]. We have found that HFD mice had reduced level of energy expenditure proteins including *β*_3_ adrenergic receptor (*β*_3_-AR), UCP2, and tyrosine hydroxylase (TH, a noradrenergic marker) in adipose tissues and the brainstem [34, 35].

Certain plants (e.g., olives, apples, and ginseng) are used as oriental medicine because of their ability to produce pharmacologically active compounds, such as pentacyclic triterpenes [36–38]. Triterpenes have demonstrated benefits in the treatment of obesity, inflammatory diseases, and cancer in humans and rodents. These compounds exert antiobesity effects by suppressing blood pressure, fat deposition, and body weight gain and increasing energy expenditure [39–41]. Increasing evidence has demonstrated the suppressive effect of pentacyclic triterpenes (oleanolic acid, ursolic acid, and betulinic acid) on visceral fat in obesity [41–43].

Bardoxolone methyl (BARD) is a synthetic pentacyclic triterpenoid compound derived from oleanolic acid. There is an increasing body of evidence showing that BARD modulates oxidative stress and inflammation via suppressing inflammatory signalling pathway and proinflammatory cytokines while activating anti-inflammatory antioxidant signalling pathway [44–46]. Moreover, BARD has been observed to benefit the immune system, brain, and peripheral tissues, including the kidneys, lungs, liver, colon, and white adipose tissue [47–51]. BARD has been used to treat obesity in humans and obese mice [49, 52]. Although BARD has been shown to induce heart failure in chronic kidney disease patients [53], this compound and its analogs are effective in obese mouse models and humans [52, 54]. Previously, we have reported the benefits of BARD in preventing obesity-associated complications (cognition and inflammation) in the brain and peripheral tissue of HFD-fed mice [34, 35, 50, 55]. In white fat, particularly, oral BARD prevents fat deposition and invasion of macrophages, inflammatory cytokines, and stress activated proteins in epididymal adipose tissue [35]. Moreover, reports from previous study and our research have indicated the involvement of BARD in energy regulation [34, 35, 49].

We have shown that HFD induces changes in metabolic and inflammatory processes in epididymal adipose tissue of mice and that BARD improves this tissue [35]. However, it has been mentioned that mesenteric adipose tissue has responded differently to HFD compared with epididymal adipose tissue [56]. Additionally, specific effects of BARD on mesenteric adipose tissue are not clear; we continue to investigate the health benefits of oral BARD in this tissue of HFD-fed mice. The outcome of this study will provide more information on understanding the pharmacological benefits of BARD in preventing obesity-related disorders through multiple effects on adipose tissue, which has been mentioned in our previous reports [34, 35].

## 2. Materials and Methods

### 2.1. Experimental Procedures on Animals

The experimental procedures on animals were assessed and approved by the Animal Ethics Committee of the University of Wollongong, NSW, Australia. Male C57BL/6J mice were obtained from the Animal Resource Centre, Perth, WA, Australia. After 1 week of acclimatisation to the institutional animal facility (temperature 22°C, 12 h light/dark cycle), the animals were divided into three groups (*n* = 7): (1) mice fed a normal diet (LFD group) (Vella Stock Feeds, Doonside, NSW, Australia); (2) mice fed a high-fat diet (HFD group) (40% fat) (SF11-095, Speciality Feeds, WA); and (3) mice fed the same HFD and supplemented with BARD in drinking water (10 mg/kg body weight) (HFD/BARD group). The dose and oral administration of BARD were chosen based upon previous studies [49, 57]. We found that HFD mice administered BARD had smaller body weight compared with HFD control as per our previous report [35]. After 21 weeks, all mice were euthanized using CO_2_ asphyxiation. Part of the mesenteric fat mass in each animal was collected and stored at −80°C for Western blot analysis. Another portion of the mesenteric fat depot was fixed in 4% paraformaldehyde and embedded in paraffin for the determination of morphology and immunohistochemistry.

### 2.2. Histology and Immunohistochemistry

Paraffin-embedded sections of mesenteric adipose tissue were cut (4 *μ*m) and stained with haematoxylin and eosin (POCD Scientific, Artarmon, NSW, Australia). Microphotographs (×40) were then taken of the stained sections using a Leica microscope. The size and number of mesenteric adipocytes were quantified by ImageJ 1.46r software (National Institute of Health, Bethesda, MD, USA), which is based upon our previous reports [34, 35].

Immunohistochemistry was used to determine the density of macrophages including total (F4/80), inflammatory (CD11c), and anti-inflammatory (CD206) phenotype in mesenteric adipose tissue. Method is based upon our previous report [35]. Briefly, paraffin-embedded sections of the mesenteric fat were cut at 4 *μ*m and antigens retrieved in sodium citrate buffer (10 mM, pH 6.0) and processed for immunohistochemical staining. Sections were probed with either anti-F4/80 (ab6640), anti-CD11c (ab33483), or anti-CD206 (ab64693). Secondary antibodies are rabbit anti-rat IgG biotinylated secondary antibody (ab6733), goat anti-Armenian Hamster IgG H&L biotin (ab5744), or goat anti-rabbit IgG H&L biotin (ab6720). All antibodies were purchased from Abcam Inc., Cambridge, MA, USA. Microphotographs were taken using a Leica microscope (×40). The quantification of macrophages is described in our previous reports [34, 35].

### 2.3. Western Blot Analysis

We used Western blot to detect the expression of different proteins including inflammatory cytokines and energy expenditure proteins following the technique reported in a previous study of ours [58]. Mesenteric adipose tissue was homogenised in NP-40 lysis buffer supplemented with protease inhibitor cocktail, *β*-glycerol phosphate, and phenylmethanesulfonyl fluoride (PMSF). The protein concentration of the extracted lysates was determined using the BCA Protein Assay (Pierce Chemical Co., Rockford, IL, USA). Homogenates (25 *μ*g protein) were dissolved in Laemmli buffer with 5% 2-mercaptoethanol, resolved on 4–12% Bis Tris-HCl gels, and transferred to polyvinylidene difluoride (PVDF) membranes (Bio-Rad Laboratories, Gladesville, NSW, Australia). The membranes were blocked in 5% bovine serum albumin before incubation with primary antibodies overnight at 4°C. The primary antibodies used were IL-1*β* (sc-7884), TNF-*α* (sc-8301), *β*_3_-AR (sc-1473), UCP2 (sc-6525), PGC-1*α* (sc-13067), pJNK (sc-6254), and pAkt1/2/3 (sc-33437) from Santa Cruz Biotechnology (Dallas, TX, USA); hydroxylase phosphoSer 40 (AB5935), tyrosine hydroxylase (AB9983), and actin (MAB1501) from Merck Millipore (Kilsyth, Victoria, Australia); I*κ*B-*α* (#9242), pSTAT3 (#9145), pERK1/2 (#4370), AMPK*α* (#2532), and phospho-AMPK*α* (#2535) from Cell Signaling Technology (Beverly, MA, USA). The following day samples were incubated with the appropriate secondary antibody for 1 hour, which were one of the following: goat anti-rabbit (AP307P) and goat anti-mouse (AP308P) from Chemi-Con International, Inc. (Temecula, CA, USA), and donkey anti-goat (sc-2033) from Santa Cruz Biotechnology (Dallas, TX, USA). Protein bands were visualised using enhanced chemiluminescence and quantified using Quantity One software (Bio-Rad Laboratories, Hercules, CA, USA) with normalisation to *β*-actin.

### 2.4. Statistical Analysis

The SPSS 19 package (SPSS, Chicago, IL, USA) was used for data analysis. One-way analysis of variance (ANOVA) test followed by a least significant difference (LSD)* post hoc* analysis was used to analyse adipose tissue histology, F4/80 macrophage density, proinflammatory cytokines, and energy expenditure proteins. All values were performed as mean ± SEM. Statistical significance was considered at *p* < 0.05.

## 3. Results

### 3.1. BARD Prevents Mesenteric Fat Deposition in HFD-Fed Mice

**Figure 1:**
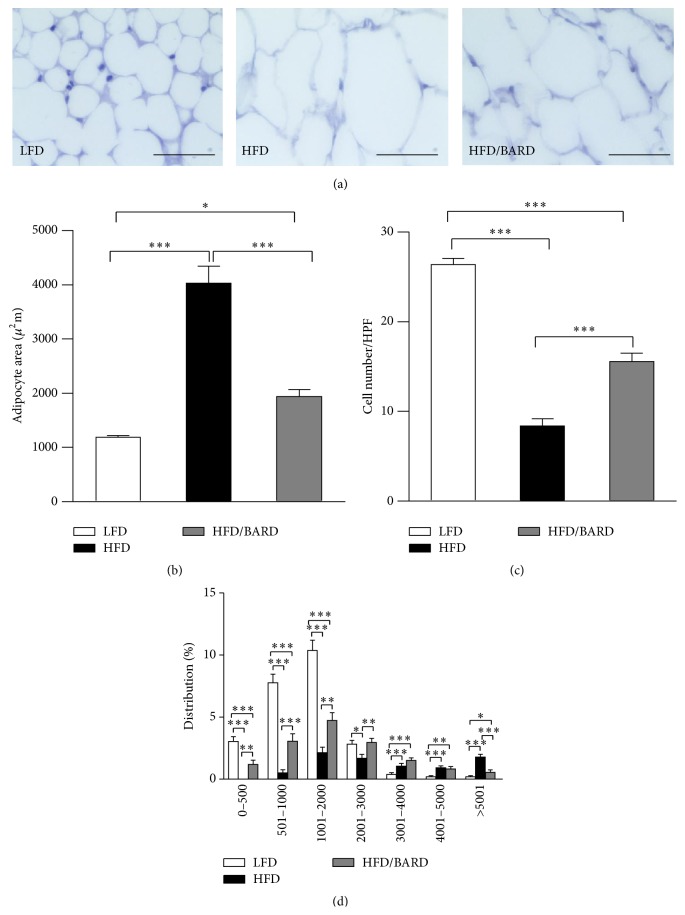
Effect of bardoxolone methyl (BARD) on mesenteric fat deposition of mice fed low-fat diet (LFD), high-fat diet (HFD), and high-fat diet supplemented with BARD (HFD/BARD). (a) Haematoxylin and eosin stained sections (×40). Bar = 50 *μ*m. (b) Mesenteric adipocyte area. (c) Number of cells per high power field (HPF). (d) Distribution of adipocyte size. All data are presented as mean ± SEM. ^*∗*^ *p* < 0.05; ^*∗∗*^ *p* < 0.01; ^*∗∗∗*^ *p* < 0.001.

We examined the effect of BARD on the size and number of mesenteric adipocytes in HFD mice (Figure 1(a)). HFD mice had threefold increase in mesenteric adipocyte size (*p* < 0.001) compared with LFD mice (Figure 1(b)). However, BARD supplementation during HFD induced twofold decrease in the size of mesenteric adipocytes (*p* < 0.001) compared with HFD control. In contrast to the increased cell surface area, the number of adipocytes was significantly reduced by three times (*p* < 0.001) in HFD mice compared with LFD mice (Figure 1(c)). Compared with HFD mice, the number of mesenteric adipocytes in HFD/BARD was doubled (*p* < 0.001). Additionally, Figure 1(d) demonstrates a shift in adipocyte size distribution in HFD-fed mice towards a smaller size with BARD treatment. The size distribution of adipocytes was similar between HFD/BARD and LFD mice. This data provides evidence that BARD prevents fat deposition in mesenteric adipose tissue.

### 3.2. BARD Prevents Infiltration and Recruitment of Inflammatory Macrophages in Mesenteric Adipose Tissue of HFD Fed Mice

**Figure 2:**
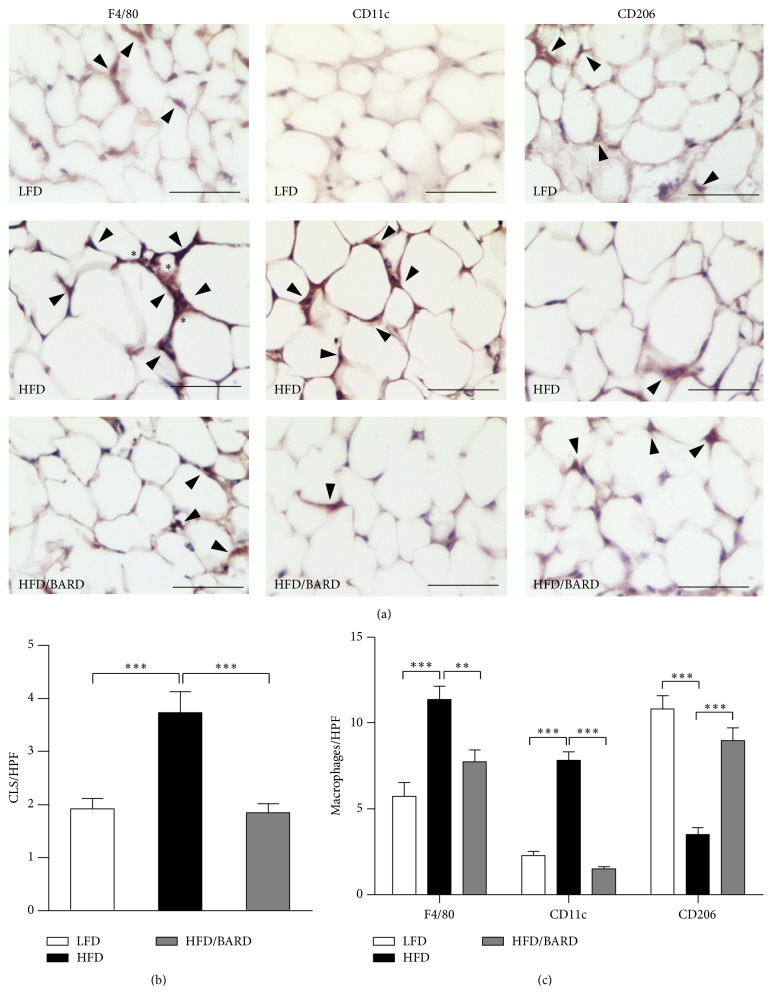
Effect of bardoxolone methyl (BARD) on expression of F4/80, CD11c, and CD206 macrophages in mesenteric adipose tissue of mice fed low-fat diet (LFD), high-fat diet (HFD), and high-fat diet supplemented with BARD (HFD/BARD). (a) F4/80, CD11c, and CD206 stained sections (×40). Bar = 50 *μ*m. The asterisks indicate F4/80 crown-like structures (CLS); the arrow heads demonstrate single F4/80, CD11c, and CD206 positive stained cells. (b) Number of CLS per high power field (HPF). (c) Number of F4/80, CD11c, and CD206 positive cells per HPF. All data are presented as mean ± SEM. ^*∗∗*^ *p* < 0.01; ^*∗∗∗*^ *p* < 0.001.

To investigate anti-inflammatory effect of BARD, we initially examined the presence of infiltrating macrophages (F4/80) by immunohistochemistry (Figure 2(a)). Compared with LFD mice, HFD mice had a marked increase in the number of F4/80 crown-like structures (+95%; *p* < 0.001), which was effectively prevented by BARD (−50%; *p* < 0.01) (Figure 2(b)). Similarly, the number of F4/80 interstitial macrophages was significantly higher in HFD mice by 98% (*p* < 0.001) compared with LFD mice and by 32% (*p* < 0.01) compared with HFD/BARD mice (Figure 2(c)). In Figure 2(c), the density of inflammatory (M1) and anti-inflammatory (M2) macrophage phenotype was also identified, assessed by the positive staining of CD11c and CD206 cells. HFD mice had threefold increase in number of CD11c positive macrophages compared with LFD mice (*p* < 0.001). The number of these cells was reduced by five times when the HFD mice were administered BARD (*p* < 0.001). In contrast, HFD mice had reduced number of CD206 positive macrophages by 67% (*p* < 0.001) compared with LFD mice. HFD mice treated with BARD had 155% increase in number of CD206 positive cells (*p* < 0.001). BARD suppresses the infiltration and recruitment of inflammatory macrophages, suggesting an anti-inflammatory effect.

### 3.3. BARD Suppresses Proinflammatory Cytokines and Oxidative Stress Proteins in Mesenteric Adipose Tissue of HFD-Fed Mice

**Figure 3:**
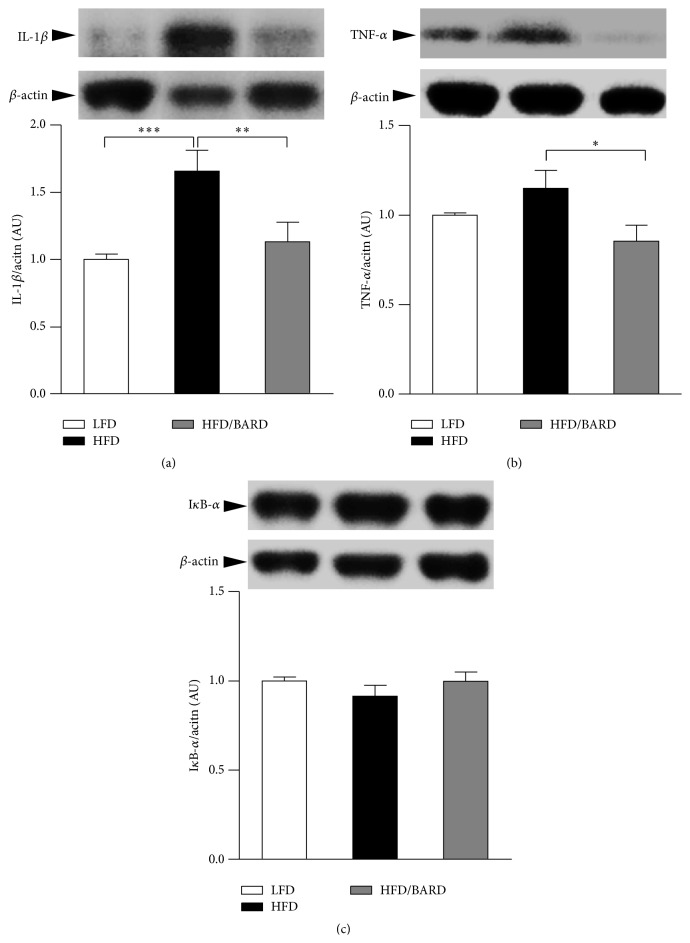
Effect of bardoxolone methyl (BARD) on expression of proinflammatory cytokines in mesenteric adipose tissue of mice fed low-fat diet (LFD), high-fat diet (HFD), and high-fat diet supplemented with BARD (HFD/BARD). (a) Representative blots and protein level of IL-1*β*. (b) Representative blots and protein level of TNF-*α*. (c) Representative blots and protein level of I*κ*B-*α*. All data are presented as mean ± SEM. ^*∗*^ *p* < 0.05; ^*∗∗*^ *p* < 0.01; ^*∗∗∗*^ *p* < 0.001. IL-1*β*: interleukin-1 beta, TNF-*α*: tumour necrosis factor alpha, I*κ*B-*α*: nuclear factor of kappa light polypeptide gene enhancer in B-cells inhibitor alpha, and AU: arbitrary unit.

Figure 3(a) shows that HFD mice had increased level of IL-1*β* protein (+66%; *p* < 0.001) compared with LFD mice. This protein was reduced by 32% (*p* < 0.01) when the HFD mice were supplemented with BARD. While a small but not statistically significant increase in TNF-*α* was also observed in HFD mice compared with LFD mice (+15%; *p* = 0.19), BARD supplementation reduced the expression of TNF-*α* by 26% (*p* < 0.05) compared with HFD mice (Figure 3(b)). Nuclear factor of kappa light polypeptide gene enhancer in B-cells inhibitor, alpha, or I*κ*B-*α* (which is negatively associated with inflammation) was also determined in the present study but no significant difference was found (Figure 3(c)). The reduced expression of proinflammatory cytokines in the present study further suggests an anti-inflammatory effect of BARD.

**Figure 4:**
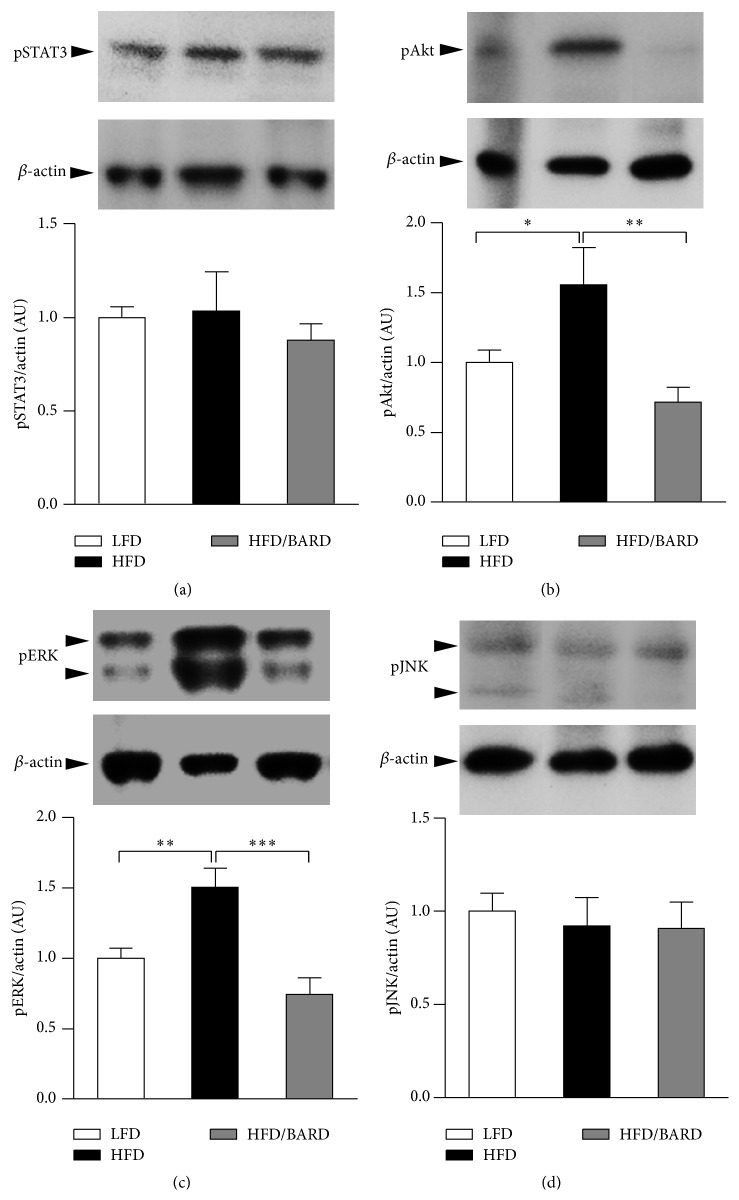
Effect of bardoxolone methyl (BARD) on expression of stress activated proteins in mesenteric fat tissue of mice fed low-fat diet (LFD), high-fat diet (HFD), and high-fat diet supplemented with BARD (HFD/BARD). (a) Representative blots and protein level of pSTAT3. (b) Representative blots and protein level of pAkt. (c) Representative blots and protein level of pERK. (d) Representative blots and protein level of pJNK. All data are expressed as mean ± SEM. ^*∗*^ *p* < 0.05; ^*∗∗*^ *p* < 0.01; ^*∗∗∗*^ *p* < 0.001. pSTAT3: phosphorylated signal transducer and activator of transcription 3, pAkt: phosphorylated protein kinase B, pERK: phosphorylated extracellular signal-regulated kinase, pJNK: phosphorylated c-Jun N-terminal kinase, and AU: arbitrary unit.

In this study, we examined the effect of BARD on oxidative stress signalling by assessing the stress activated proteins including phosphorylated protein kinase B (pAkt), phosphorylated extracellular signal-regulated kinase (pERK), phosphorylated signal transducer and activator of transcription 3 (pSTAT3), and phosphorylated c-Jun N-terminal kinase (pJNK) in mesenteric adipose tissue. In Figure 4(b), mice fed a HFD had 56% increase in pAkt compared with those fed a LFD (*p* < 0.05). On the other hand, BARD administration induced 54% reduction in pAkt protein (*p* < 0.01). As shown in Figure 4(c), HFD mice had a significant increase in pERK by 50% (*p* < 0.01) compared to LFD mice. However, BARD reduced pERK by 50% (*p* < 0.001) in HFD mice. There were no statistical differences in pSTAT3 and pJNK among three groups of mice (Figures 4(a) and 4(d), resp.). These data demonstrate that BARD prevents the increase of pAkt and pERK which occurs in HFD-fed mice, suggesting that BARD suppresses oxidative stress-induced by HFD.

### 3.4. BARD Enhances Energy Metabolism in Mesenteric Adipose Tissue of HFD-Fed Mice

**Figure 5:**
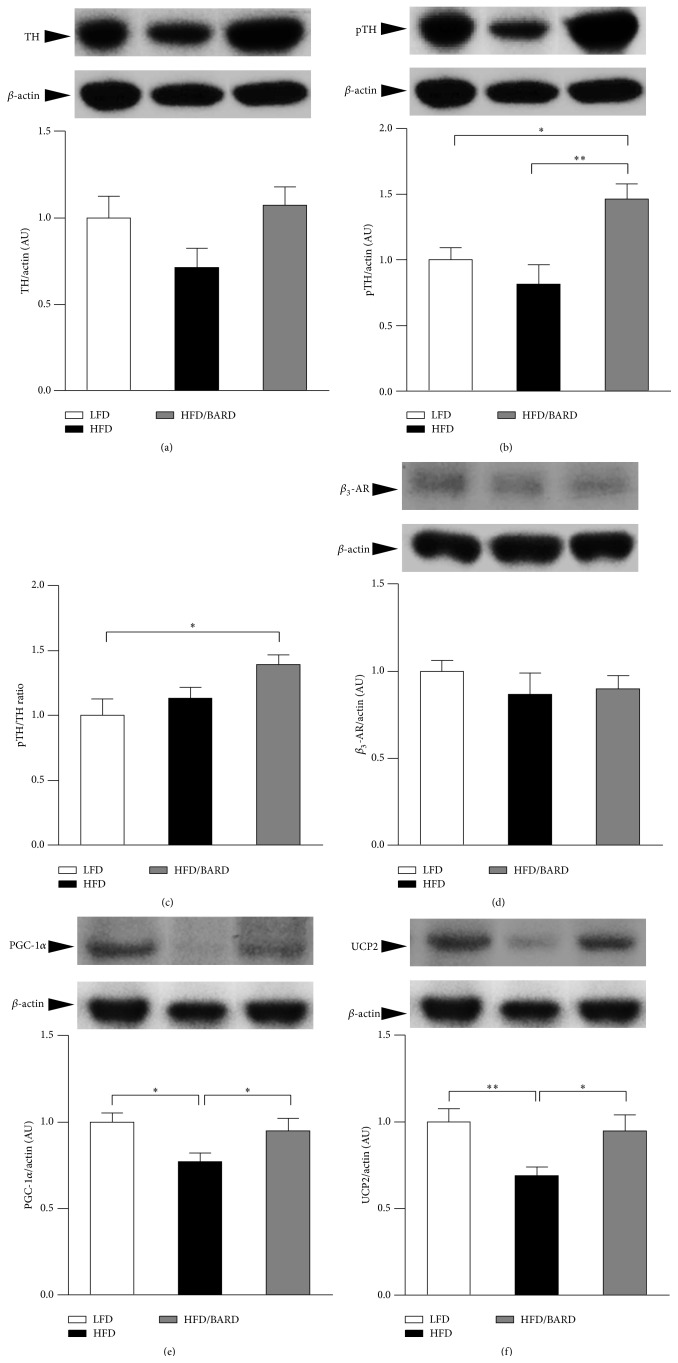
Effect of bardoxolone methyl (BARD) on expression of rate limiting enzyme, *β*_3_-AR, PGC-1*α*, and UCP2 in mesenteric adipose tissue of mice fed low-fat diet (LFD), high-fat diet (HFD), and high-fat diet supplemented with BARD (HFD/BARD). (a) Representative blots and protein level of TH. (b) Representative blots and protein level of pTH. (c) pTH/TH ratio. (d) Representative blots and protein level of *β*_3_-AR. (e) Representative blots and protein level of PGC-1*α*. (f) Representative blots and protein level of UCP2. All data are expressed as mean ± SEM. ^*∗*^ *p* < 0.05; ^*∗∗*^ *p* < 0.01. TH: tyrosine hydroxylase, pTH: phosphorylated TH; *β*_3_-AR: *β*_3_-adrenergic receptor; PGC-1*α*: peroxisome proliferator-activated receptor gamma coactivator 1-alpha; UCP2: uncoupling protein 2; AU: arbitrary unit.

We first investigated the effect of BARD on noradrenergic activation, assessed by expression of TH protein and phosphorylation. There was no significant difference in protein expression of total TH among mouse groups (Figure 5(a)). Compared with LFD- and HFD-fed mice, HFD mice administered BARD had increased level of phosphorylated TH (pTH) protein by 46% (*p* < 0.05) and 80% (*p* < 0.01), respectively (Figure 5(b)). Consistently, pTH/TH ratio in HFD/BARD mice was increased by 39% (*p* < 0.05) compared with LFD mice and by 23% (*p* = 0.08) compared with HFD mice (Figure 5(c)). The effect of BARD on adrenergic activation and mitochondrial uncoupling was also assessed via the protein expression of *β*_3_-AR and PGC-1*α* and UCP2. There was no significant difference in expression of *β*_3_-AR protein among mouse groups (Figure 5(d)). The HFD mice showed a significant decrease in protein expression of PGC-1*α* (−22%; *p* < 0.05) and UCP2 (−33%; *p* < 0.05) compared with the LFD mice (Figures 5(e) and 5(f), resp.). Compared with HFD control, HFD mice administered BARD had significant increase in PGC-1*α* protein by 24% (*p* < 0.05) and in UCP2 protein by 30% (*p* < 0.05).

**Figure 6:**
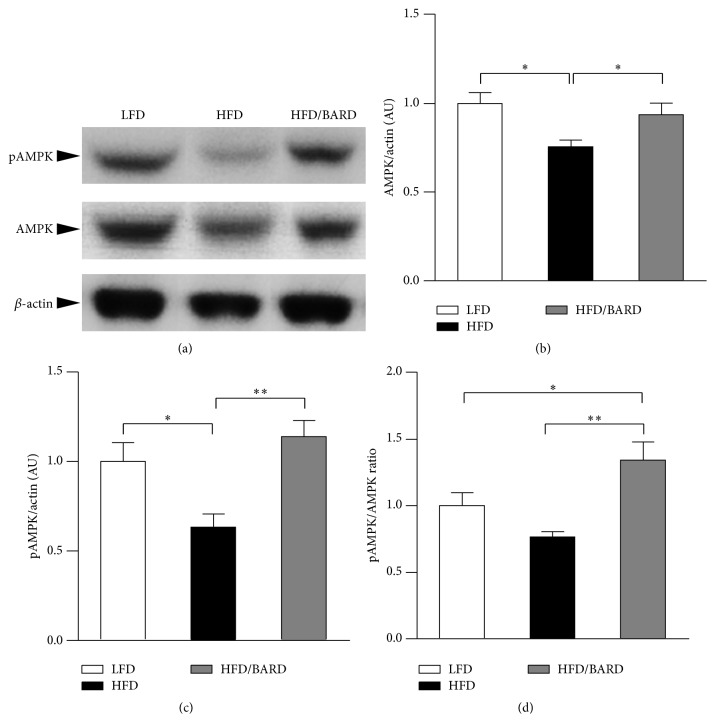
Effect of bardoxolone methyl (BARD) on expression of AMPK protein and its activity in mesenteric adipose tissue of mice fed low-fat diet (LFD), high-fat diet (HFD), and high-fat diet supplemented with BARD (HFD/BARD). (a) Representative blots. (b) Protein level of AMPK. (c) Protein level of phosphorylated AMPK. (d) pAMPK/AMPK ratio. All data are expressed as mean ± SEM. ^*∗*^ *p* < 0.05; ^*∗∗*^ *p* < 0.01. AMPK: AMP-activated protein kinase; pAMPK: phosphorylated AMPK; AU: arbitrary unit.

We also further investigated the effect of BARD on the protein expression of total AMPK and its phosphorylation (Figure 6(a)).  Figure 6(b) shows that HFD induced a significant decrease in total AMPK protein (−25%; *p* < 0.05) compared with LFD mice. However, compared with HFD mouse control, those administered BARD had significant increase in total AMPK protein by 24% (*p* < 0.05). In Figure 6(c), mice fed a HFD had reduced phosphorylated AMPK (pAMPK) protein by 37% (*p* < 0.05) compared with those fed a LFD. Compared with HFD mice, pAMPK protein in HFD/BARD mice was increased by 80% (*p* < 0.01). Similarly, HFD mice administered BARD had increased level of pAMPK/AMPK ratio by 34% (*p* < 0.05) compared with LFD mice and by 75% (*p* < 0.01) compared with HFD mice (Figure 6(d)). Taken together, these data suggest that BARD improves energy metabolism in mesenteric adipose tissue of HFD-fed mice.

## 4. Discussion

In the present study we investigated the effect of oral BARD at 10 mg/kg body weight, administered daily in drinking water on the mesenteric adipose tissue of mice fed HFD. The data demonstrate that BARD can suppress HFD induced fat deposition, inflammation, and oxidative stress, and it enhances energy metabolism in mesenteric adipose tissue.

Increasing evidences have shown that HFD induces increase of body weight and fat deposition in various tissues [56, 59] and changes in cellularity (cell size and number) are characteristic of obesity [8, 60]. Presently, we found that BARD increased the number and distribution of small adipocytes in mesenteric fat of HFD-fed mice. Studies have shown a reduction in the number of large-sized adipocytes and an increase in the number of small-sized adipocytes in adipose tissue is correlated to improved insulin sensitivity and reduced obesity-induced insulin resistance [61–63]. Consistently, we have found that BARD prevents insulin resistance in mice on HFD [55]. Similarly, the preventive effect of BARD on fat deposition in epididymal and brown adipose tissue suggests that BARD may prevent obesity-related insulin resistance and diabetes mellitus through regulation of adipocytes. Evidences have shown that BARD and its analogues reduce fat mass in obese mouse models [49, 54]. This is also consistent with the reducing effect of BARD on body weight, which was mentioned in our previous report and other studies [35, 52]. The effect of BARD is consistent with the antiobesity function of pentacyclic triterpenes [41–43]. The present study provides the first data of the effect of BARD on mesenteric fat deposition and suggests that this molecule has potential in preventing visceral obesity.

In obesity mesenteric adipose tissue becomes inflamed, demonstrated by the infiltration of immune cells (macrophages), which leads to a proinflammatory immune response (increased production of proinflammatory cytokines) [3, 64]. In the present study, BARD administration reduced the infiltration of inflammatory macrophages and proinflammatory cytokine expression in HFD-fed mice, suggesting an anti-inflammatory effect. This is consistent with its derivative oleanolic acid, which reduces F4/80 positive macrophage infiltration in the kidneys of C57BL/6 mice affected by oxidative stress [65]. In another study BARD reduced the number of infiltrating alveolar macrophages in lung tissue by 40% in bleomycin treated C57BL/6 mice [48]. Similarly, we have shown that BARD reduces M1 macrophage phenotype (CD11c) and increases M2 macrophage phenotype (CD206) in BAT of HFD-fed mice [34]. In the present study, BARD suppresses the expression of proinflammatory cytokines within mesenteric adipose tissue of HFD mice. Obesity is characterised by low-grade inflammation, reflected, in part, by increased expression of circulating (TNF-*α*) and local (TNF-*α*, IL-1*β*) proinflammatory cytokines in diet-induced obese mice [66–68]. Present data, thus, demonstrate anti-inflammatory property of oral BARD through suppression of proinflammatory cytokines. Previously, BARD treatment reduced the mRNA expression of IL-1*β*, TNF-*α*, and IL-6 in the liver and epididymal fat of diet-induced diabetic mice [49]. Since the upregulation of proinflammatory cytokines and inflammatory macrophage phenotype (i.e., CD11c) in mesenteric adipose tissues relates to the development of colitis, inflammation, and steatohepatitis in liver, insulin resistance [15, 16], it seems that BARD provides health benefits in HFD mice.

Escalated level of protein kinases including protein kinase B (Akt), extracellular signal-regulated kinase (ERK), signal transducer and activator of transcription (STAT), and c-Jun N-terminal kinase (JNK) has been observed in HFD-fed mice. We found an increased level of pAkt and pERK in HFD mice which were suppressed by BARD. Activation of Akt and ERK is linked to oxidative stress in adipocytes and in the development obesity-associated complications [69, 70]. In visceral fat, a greater protein level of oxidative stress molecules and ERK has been observed in obese women, when compared to healthy women [20]; the reduced ERK protein by BARD may indicate an antioxidative function. BARD reduces nephritis in mice through suppression of AKT/ERK/NF-*κ*B signaling [71]. BARD induces anti-inflammatory and antioxidative effects in HFD mice in our study, which is consistent with the pharmacological function of BARD and its related compounds (oleanolic acid), which induce antioxidant anti-inflammatory properties via activating antioxidant response element- (ARE-) Kelch ECH associating protein 1- (Keap1-) nuclear factor erythroid 2-related factor 2 (Nrf2) network [44, 47, 72, 73]. A study on cardiac cells has shown that ERK activation suppresses Nrf2, leading to oxidative stress-induced insulin resistance* in vitro* and* in vivo* [74]. The reducing effect of BARD on ERK and associated Akt activity, therefore, is an indicator for antioxidative stress of this compound. Previously, we have found that BARD suppresses the expression of ERK, Akt, STAT3, and JNK proteins in epididymal adipose tissue of HFD-fed mice [35]. Additionally, BARD analog, dihydro-2-cyano-3,12-dioxooleana-1,9(11)-dien-28-oic acid (CDDO)-trifluoroethyl amide (dh404), suppresses ERK activity in the hearts of streptozotocin-induced diabetic mice and diabetic patients, which is associated with the reduced oxidative stress and improved insulin sensitivity [74]. From the outcome of the present study, further research on various stress oxidative stress genes and signalling pathways will help to understand antioxidative function of BARD in diet-induced obesity.

Reduced level of TH and its activity in the brain and peripheral tissues is linked to reduced energy expenditure and obesity [31, 75, 76]. Presently, BARD induces nonradreneric activation through increasing the activity of rate limiting enzyme TH. We have found that BARD consistently increases TH signalling activity in epididymal and brown adipose tissue and the brainstem of HFD-fed mice [34, 35]. Previous study has reported that the increase of TH activity in mesenteric adipose tissue is associated with the increased catecholamine biogenesis and production in this tissue [77]. In the present study, BARD also increases PGC-1*α* and UCP2 protein in mesenteric adipose tissue of HFD-fed mice. Recent reports on human pluripotent stem cell have shown that PGC-1*α* performs as transcriptional regulator of mitochondrial biogenesis and is involved in cellular respiratory events and energy production [78, 79]. We have observed the increasing effect of oral BARD on PGC-1*α* protein in the brainstem of HFD-fed mice [34]. Additionally, increased UCP2 protein was observed in visceral fat of obesity resistant A/J and C57BL/KsJ (KsJ) mouse strains fed a HFD, and studies have described the possible role of UCP2 in energy metabolism [79–82]. Previously, mitochondrial uncoupling has been proposed as mechanistic mechanism for the antiobesity effect of ursolic acid in HFD-fed mice [83]. Similarly, we have previously reported the upregulation of UCP2 protein in epididymal adipose tissue of HFD-fed mice administered BARD [35]. An* in vitro* study on muscle cells has shown that the increased expression of PGC-1*α* protein is linked to the activation of mitochondrial uncoupling and increased oxygen consumption [84]. These data, therefore, are an indicator for the involvement of BARD in the control of energy expenditure. Moreover, we found that BARD increased total AMPK protein and its phosphorylation in mesenteric adipose tissue of HFD-fed mice. AMPK directly stimulates energy expenditure in the peripheral tissues [85–87]. Reports have implicated AMPK in energy regulation in visceral fat, and induction of this molecule results in resistance to obesity [30, 80]. Previously, BARD treatment activates AMPK in the muscle and the liver of obese mice fed HFD, which is associated with increased oxygen consumption and the amelioration of obesity in mice fed HFD [49]. From the outcome of this study, we suggest that BARD enhances energy metabolism through activation of noradrenergic and AMPK activity, mitochondrial biosynthesis, and mitochondrial uncoupling, thus providing a defence against obesity. Since evidence for the increase of energy expenditure by BARD has been previously reported [49], present study provides potential metabolism contributing to the enhancing effect of BARD on thermogenesis.

The study is the first to provide evidence that BARD regulates mesenteric adipose tissue in HFD-fed mice by preventing fat deposition and inducing the anti-inflammatory and antioxidant effect while enhancing energy metabolism. In particular, BARD administration significantly reduced the number of large-sized adipocytes while it increased the number of small adipocytes, suggesting its preventive effect on fat deposition. In addition, it reduces number of infiltrating macrophages and recruitment of M1 macrophages, which were accompanied by reduction of proinflammatory cytokines and stress activated protein, suggesting its anti-inflammatory and antioxidative stress effect of BARD. On a mechanistic level, BARD enhanced energy expenditure through increasing the activity of rate limiting enzymes TH and AMPK. This was accompanied by the induction of PGC-1*α* and UCP2 protein. The improving effect of BARD on metabolic and inflammatory processes in present study is consistent with the suppressive effect of BARD on body weight, thus it may link to its antiobesity effect. Overall, the present study suggests the potential of oral BARD in preventing visceral fat pathology in obesity. From outcome of present study and our previous reports [34, 35, 50, 55], we proposes the regulatory role of BARD in adipose tissues, which would be an important factor for body weight suppression of this compound. Further studies will help not only to clarify our observations but also to warrant further applications of BARD in humans who are susceptible to obesity.
